# Recent Progress in Voice-Based Sasang Constitutional Medicine: Improving Stability of Diagnosis

**DOI:** 10.1155/2013/920384

**Published:** 2013-08-26

**Authors:** Jun-Su Jang, Young-Su Kim, Boncho Ku, Jong Yeol Kim

**Affiliations:** KM Health Technology Research Group, Medical Research Division, Korea Institute of Oriental Medicine, 1672 Yuseongdae-ro, Yuseong-gu, Daejeon 305-811, Republic of Korea

## Abstract

Sasang constitutional medicine is a unique form of tailored medicine in traditional Korean medicine. Voice features have been regarded as an important cue to diagnose Sasang constitution types. Many studies tried to extract quantitative voice features and standardize diagnosis methods; however, they had flaws, such as unstable voice features which vary a lot for the same individual, limited data collected from only few sites, and low diagnosis accuracy. In this paper, we propose a stable diagnosis model that has a good repeatability for the same individual. None of the past studies evaluated the repeatability of their diagnosis models. Although many previous studies used voice features calculated by averaging feature values from all valid frames in monotonic utterance like vowels, we analyse every single feature value from each frame of a sentence voice signal. Gaussian mixture model is employed to deal with a lot of voice features from each frame. Total 15 Gaussian models are used to represent voice characteristics for each constitution. To evaluate repeatability of the proposed diagnosis model, we introduce a test dataset consisting of 10 individuals' voice recordings with 50 recordings per each individual. Our result shows that the proposed method has better repeatability than the previous study which used averaged features from vowels and the sentence.

## 1. Introduction 

Sasang constitutional medicine (SCM) is a unique form of tailored medicine in traditional Korean medicine. The origin of SCM was started by Lee, a Korean medical doctor [1]. In SCM theory, human beings can be categorized into one of the four Sasang constitutional (SC) types (Tae-Yang: TY, Tae-Eum: TE, So-Yang: SY, and So-Eum: SE) according to their inherited characteristics, such as temperament profile, physiological and pathological features, susceptibility to particular diseases, and responses to drugs [2, 3]. In the principle of SCM, treatment is to recover balance between hypoactive organs and hyperactive organs, based on one's constitution. Therefore, determining one's SC type is important and many attempts have been made for standardization of SC diagnosis [4–6].

Diagnosis using voice is one of the important parts in SCM. The relation between SC types and voice is referred in the literatures [1, 7]. TY type's voice is resonant, clear, and loud. It was derived from good respiratory organs. TE types have a load baritone voice, which sounds thick, heavy, and grave. SY types have clear, fast, and high-pitched voice. They are talkative, hasty, and vigorous. SE type's voice is clam and easy. It sounds gentle, slow, and lively. Some of the voice characteristics are described by using utterance quality and style, but personality term is also used to depict voice characteristics. Since the vague linguistic representation can be understood differently to each oriental medicine doctor, an objective and standardized voice diagnosis is needed. 

To establish objective and scientific diagnosis of SC types, many studies used computerized voice analysis method. Early studies focused on finding correlation between SC types and various voice features such as fundamental frequency, formants, and energy of voice signal [8–10]. In 2004, Park and Kim found a significant difference between SE and SY in formant frequency and formant bandwidth [11]. Their result showed that SY voice was clearer than SE voice, which was coincident with the SCM literatures. Kim et al. developed a voice analysis system, namely, phonetic system for Sasang constitution (PSSC), and applied it to investigate constitutional characteristics of Korean adult males and females [12, 13]. They used pitch, amplitude perturbation quotient, shimmer, octave, and energy as voice features. Choi et al. studied characteristics of the Korean adult male sound using PSSC with a sentence [14]. In 2009, Kang et al. analysed 144 voice features from 5 vowels and one sentence recorded from 473 people [15]. They developed a constitutional classification method using support vector machine; however, their classifier was able to apply correctly in only limited voice data [16].

Although many studies existed, they were not sufficient for practical use, because the diagnosis model was made by using a limited number of data gathered in few sites. To overcome the generalization problem, a set of large data, collected from 23 different oriental clinics, were established in the study of Do et al. [4]. They developed an integrative SC analysis tool (SCAT) using four individual diagnosis components: face, body shape, voice, and questionnaire. They trained four individual diagnosis models using logistic regression method, and the four models were combined into a final integrated model. They evaluated the integrated model to a test dataset which was not used in training stage, for confirming generalization ability. As a generalization test result, the diagnostic accuracies of the integrated model were 64.0% and 55.2% in the male and female patient groups, respectively. The accuracies of the voice component were 39.9% and 37.5% for male and female, respectively. Although the accuracies of voice component were low, voice information was still helpful to increase the performance of the integrated model. Another research using the same voice data of Do et al. was carried out by Kim et al. [5]. They employed linear discriminant analysis as a classifier, and the training accuracies were 51% and 47% for male and female, respectively. However, they only provided the training result, which is generally higher than generalization test result. 

Most of the previous studies were tried to find voice features that were strongly correlated to SC types and developed SC classification model. A few weakly correlated features were found, and different types of classifier were developed; however, none of those studies mentioned the diagnostic stability of their method. Because of the natural variation in speech, diagnosis results can be different for the same individual speaker. To reduce a lot of unsystematic variation, speaker's utterance should be constrained by a strict standard operating procedure (SOP). Kim et al. studied about developing SOP for extracting stable voice features that can characterize individual's voice quality consistently [17] and also analysed stable voice features [18]. 

In this paper, we propose a method to improve stability of voice-based SC diagnosis. All of large database, strict SOP, stable voice features, and a robust classification method are required to obtain stability of diagnosis. We use the same voice data as in the previous study [4], since they are known as the largest data containing patients' SC type proved by herbal remedy [19]. The proposed method uses only sentence recordings, against many previous studies using vowels or both vowels and sentences. Most of recent speaker identification studies use words or sentences, rather than vowels, since words or sentences are better to distinguish each individual's voice characteristics [20, 21]. Our results also show that the detailed analysis using a sentence recording has better repeatability in SC classification than the previous approach [4], which used vowels and the sentence.

## 2. Materials and Methods

### 2.1. Voice Data for Sasang Constitution Diagnosis

Voice data were collected from 23 oriental medical clinics. The patient whose SC type was diagnosed to TY was excluded due to its small sample size compared with other three SC types. The total number of patients used in this study was 1,969, ranging in age 15–60 years in both genders. The patients did not suffer from any voice-related disease so that they could speak naturally with their own voice quality. Their SC type was examined by SCM practitioners, who had more than five years of experience in clinical practice. A more detailed procedure of data collection was described in Song et al. [19]. Original database included face, body shape, and questionnaire information; however, only voice data were considered in this study.

Recording environment and procedure was strictly controlled by an SOP. Environment noise kept below 40 dB for low-noise recording. Recording room temperature was controlled to 20°C ± 5°C, and humidity kept to 40% ± 5%. Sound Blaster Live 24 bit External Soundcard and Sennheiser e-835s Microphone with a microphone stand were equipped. The distance between the patient and microphone was 4–6 cm. Recording systems were controlled to avoid echoes of voice or irregular resonance. Recording was saved as a wav file with setting of mono 16 bit integer and 44.1 kHz sampling frequency. 

Voice data consisted of five vowels (/a/, /e/, /i/, /o/, and /u/) and a sentence. The sentence was recorded twice. Since the all patients were Korean, the sentence was also composed of Korean words. As voice features should represent the natural characteristics of patients in short recordings, the patients were asked to pronounce their natural voice without tension as possible. Before actual recording, an operator instructed the patients about the recording contents and allowed them to rest for 1 hour. Each vowel was uttered at least 3 s. The sentence was uttered in their ordinary speed and tones. In this paper, we used only sentence part to diagnose constitution, and for comparison, the previous study [4] used both five vowels and sentence.

### 2.2. Voice Feature Extraction

**Figure 1:**
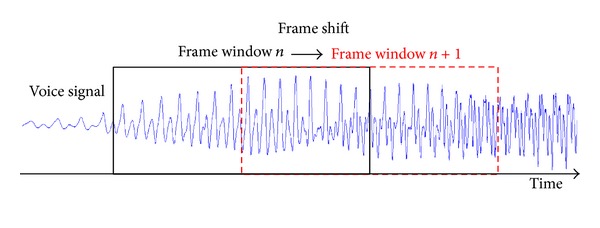
Frame window shift for extracting voice features for each frame.

Figure 1 shows how voice features are calculated from a voice signal. We should define the size of a frame window, which is the minimum length to process. Then, we can extract voice features in the frame. After processing the current frame window, the same feature extraction is applied to the next frame window. In this study, the size of a frame window was 46.4 ms, which mapped to 2^11^ audio samples in 44.1 kHz sampling frequency. Neighboring frame windows were overlapped by 50%. 

There exist more than a hundred valid frames in each vowel or sentence. In the past studies, there were many candidate voice features, such as fundamental frequency (*F0*), formants, and jitter. Most of them were calculated by averaging the feature values obtained from the all valid frames in the voice signal. An averaged feature could be good for monotonic utterance like a vowel. For example, *F0* for a vowel is calculated by averaging all *F0* values from the valid frames. The vowel utterance is monotonic, which means that every single *F0* value from each frame should be similar. Therefore, averaging is appropriate for characterizing *F0* for a vowel. Most of the previously used features from a vowel are averaged features, which may represent quality of the vowel properly. 

However, features from the vowel are not proper to characterize individual's stable voice quality. It can be easily verified that words or sentences are more suitable for characterizing individual's stable voice quality in the most of recent speaker identification application [20, 21]. To make a stable diagnosis model, which has a good repeatability for the same individual, we focus on the sentence rather than vowel. In contrast to the features for vowel, averaged features are hardly used for the sentence since the features vary a lot in many frames. When averaging, we may lose useful information of individual's voice characteristics. Therefore, we analyse the features from every single frame. 

Let **y**_*i*_ be the feature vector extracted from the *i*th valid frame. Then, we define the feature vector as follows:

(1) $$\begin{array}{c} y_{i}=\left[\begin{array}{c} x_{i}\\ {ts}_{i} \end{array}\right], \end{array}$$

where *ts*_*i*_ indicates the relative position of the *i*th frame in all valid frames, and it has a range between 0 and 1. When the feature vector comes from the first frame of the sentence, the value of *ts*_*i*_ is 0. The vector **x**_*i*_ is a column vector of candidate voice features. We use Mel-frequency cepstral coefficients (MFCCs) as the candidate voice features. MFCCs are coefficients of the short-term power spectrum of a sound, based on a linear cosine transform of a log power spectrum on a nonlinear mel scale of frequency [22]. The mel scale approximates the human auditory system's response more closely than the linearly spaced frequency bands. MFCCs are widely used in speech and speaker recognition systems [23]. Total 12 MFCCs were used in our study. 

Finally, feature vector **y**_*i*_ is a 13-dimensional vector, and the total number of **y**_*i*_ is the same as the number of all valid frames, which is usually more than a hundred for a sentence. By defining the feature vector including relative position of the frame, each feature vector contains not only voice characteristics of a frame signal, but also the information where the voice features come from. The feature vector was extracted using C++ program combined with HTK [24].

Since the voice features showed nonlinear fluctuation according to the age in general, a process to reduce the effect of age was required. To eliminate the age effect to the voice features, a standardization process was performed the same as Do et al. [4]. All MFCCs were standardized by using their moving averages and standard deviations derived from the data within the length of age ±5 years for the specific age.

### 2.3. Gaussian Mixture Model-Based Classification

The problem of SC diagnosis is treated as three-class classification, since TY type is usually excluded due to its rareness. Many statistical pattern recognition methods were applied to SC classification, such as logistic regression [4], linear discriminant analysis [5], and support vector machine [16]. Although many attempts were made in the past, none of them analysed the features from each frame separately. We propose a detailed analysis method to deal with the features of each frame using Gaussian mixture model (GMM). It is widely used for speech analysis area, since it can effectively model voice characteristics. GMM is denoted as

(2) $$\begin{array}{c} p\left(y\;|\;\lambda \right)=\sum_{j=1}^{M}w_{j}\cdot g\left(y\;|\;\mu_{j},\Sigma_{j}\right), \end{array}$$

where *λ* is one of the SC classes, *M* is the total number of Gaussian models, *w*_*j*_ is the weight for the *j*th Gaussian model, and *g*(**y** | ***μ***_*j*_, Σ_*j*_) is Gaussian function with mean ***μ***_*j*_ and covariance matrix Σ_*j*_.

**Figure 2:**
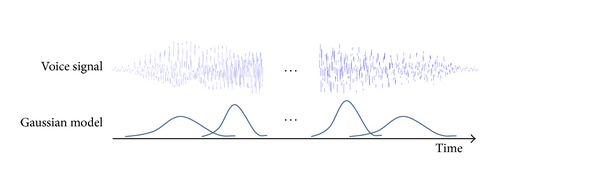
Gaussian mixture model along the time axis to cover each part of the voice signal.

The basic idea of using GMM for SC classification is that we put each Gaussian model along the time axis to cover each part of the voice signal. Since we have to deal with a few feature vectors from all valid frames, it is important to use the relative position of each feature vector. In other words, someone's voice features extracted from a certain part of the sentence should be compared to a model that is generated by voice features extracted from similar parts. The feature vector **y**_*i*_ has 12-dimensional MFCCs and relative position information as the 13th element. Hence, each Gaussian model in GMM is 13-dimensional, and we can display the location of each Gaussian model along the time axis by using the 13th element.  Figure 2 shows that GMM covers each part of the voice signal.

Since GMM is needed for each gender and SC type, total 6 groups of feature vectors extracted from training data are prepared for making 6 GMMs. GMM is trained for each feature vector group using EM algorithm [25]. In training process, the feature vectors need to be assigned to one of the Gaussian models as an initial assignment. The initial assignment can be done by uniformly dividing the feature vectors using the *ts*_*i*_ value. For example, when the number of Gaussian model is 15 (*M* = 15), the feature vectors are extracted from the first 1/15 part of the sentence assigned to the first Gaussian model. This is just for initial assignment, and the assignment is automatically changed in learning process.

After GMM for each SC class is trained, we follow the basic maximum likelihood-based classification. Probability that input recording falls into class of *λ* is calculated as follows:

(3) $$\begin{array}{c} \overset{-}{p}\left(y\;|\;\lambda \right)=\prod_{i=1}^{N}p\left(y_{i}\;|\;\lambda \right). \end{array}$$

The total number of feature vectors is *N*, and the *p*(**y**_*i*_ | *λ*) is calculated using (2) for each feature vector. Then, the estimated probability for being in each SC type *π*_*k*_ can be denoted as

(4) $$\begin{array}{c} \pi_{k}=\frac{\overset{-}{p}\left(y\;|\;\lambda_{k}\right)}{\sum_{l=1}^{3}\overset{-}{p}\left(y\;|\;\lambda_{l}\right)}. \end{array}$$

The index *k* ∈ {1,2, 3} indicates SC types, TE, SE, and SY, respectively. Finally, let *h* ∈ {1,2, 3} be the predicted SC types; then the classification rule for SC types using *π*_*k*_ is given by

(5) $$\begin{array}{c} h=\underset{k\in \{1,2,3\}}{\text{argmax}}\left(\pi_{k}\right). \end{array}$$

This means that simply taking the class of maximum probability is the final decision.

## 3. Experimental Results

### 3.1. GMM-Based Classifier

Total 1,969 samples (1,263 females and 706 males) were used to train GMMs. Since the voice characteristics were different according to gender, GMMs were separately trained for each gender. Total 6 GMMs, representing 3 types of SC for each gender, were obtained. Each model had 15 Gaussians to cover the voice signal of a sentence.  Table 1 shows the location of each Gaussian in time axis by summarizing the mean value of *ts*_*j*_. The value of *ts*_*j*_ indicates the relative position of the *j*th Gaussian model. For example, the first Gaussian in female TE has the relative position value of 0.05. This means that the first Gaussian is located in the first 5% part of the sentence, and input feature vectors are extracted around 5% part of the sentence are mainly judged by the first Gaussian.

Although the purpose of this study is to improve repeatability of diagnosis, we shortly mention the accuracy of the classifier. To make a fair comparison, we used the same test dataset of previous study conducted by Do et al. [4]. The accuracies of the GMM-based classifier were 41.3% and 39.3% for male and female, respectively, which are slightly better than Do et al. It should be noted that the accuracies of voice diagnosis are still low and have to be improved further. However, improvement of accuracy is not the main point of this study. The proposed method has better repeatability (see the next section) than the previous study while maintaining the similar accuracy.

### 3.2. Diagnosis Repeatability Comparison

To evaluate the repeatability of the SC classification, we gathered voice data of 10 individuals. Total 50 recordings for each individual were prepared with the same way of gathering the training data explained in Section 2.1. It took more than a week to record 50 times for each individual, so that the recordings contained the intrapersonal voice variations enough. We used this data to compare the proposed method and the previous study conducted by Do et al.

We tested the repeatability and probability values (mean and standard deviation) as a measure of diagnosis stability. Repeatability was defined by the ratio of the number of majority decision to total number of tests. In every test, we also obtained the probability value of the current decision, that is, the maximum probability among *π*_*k*_. Since the problem of SC diagnosis is three-class classification, the maximum probability is larger than 1/3. A stable classification has a high percentage of repeatability and a low probability standard deviation.

Table 2 shows the comparison results of diagnosis stability. For 10 individuals, the average repeatability of the proposed method was 91.6%, which clearly showed better repeatability than 78.2% of Do et al. And probability standard deviation was 0.022, which was much lower than 0.102 of Do et al. It means that the proposed method generated more consistent results in 50 times classification test of the same individual's voice than the previous study. Interpretation of the probability mean value might be controversial. However, we think that a low probability mean value is more appropriate, considering that the accuracy of the voice diagnosis is low around 40%.

Some of subjects, such as subjects 6 and 8, had low repeatabilities in our method. The subject whose voice feature vector is located close to decision boundary can have a low repeatability, because a small change of the feature vector can switch the final decision. In this case, the probability value is also close to 1/3, which means that the decision has less confidence. We may have a difficulty in determining one's constitution with high confidence when his/her voice does not have clear constitutional characteristics. This can drop the repeatability of diagnosis. However, even in this case, it is good to have similar probability values in repeated tests for the same subject. Our method shows stable diagnosis in probability sense, having low probability standard deviations even in low repeatability cases.

An improved repeatability is obtained from the proposed method that analyses every single frame of the sentence. The improvement is caused by not only analysis method but also recording contents. Our approach uses only sentence rather than both vowels and the sentence since the features from vowels have relatively large variations for the same subject. To examine the stability of each feature value itself, standard deviations of feature values from the sentence and vowels are summarized in Table 3. Some of the vowel features, used in the study of Do et al., are compared to the sentence features in this study. Table 3 shows that sentence features have smaller standard deviations than vowels. Standard deviations of the most sentence features are ranged from 0.2 to 0.7, while standard deviations of many vowel features are larger than 1. Therefore, stability of diagnosis with only sentence will outperform other cases, regardless of classification algorithms.

## 4. Discussion and Conclusions

In this study, a stable classification method for voice-based Sasang constitutional diagnosis was proposed. In contrast to the previous study, which used averaged features from vowels and one sentence, the proposed method used MFCCs extracted from the sentence only. Since we did not use averaged features to avoid losing useful information of individual's voice characteristics, features from every valid frame were required to be analysed separately. We defined a feature vector that contained MFCCs and relative position of the current frame in the voice signal. Therefore, the feature vector represented both voice features and information about where the features were extracted in time axis of the voice signal. This technique was necessary to use the sentence instead of the vowels, because the sentence included a variety of voice features in each frame, which should not be averaged.

The proposed method employed GMM for detailed analysis of every valid frame in voice signal. Each Gaussian probability model represented constitutional and individual characteristics by covering each part of the sentence. An input feature vector extracted from a certain part of the sentence could be mapped to Gaussian models that were trained using voice features extracted from similar parts of training data. Our method was compared to the previous study, which used both vowels and the sentence. The proposed method had an average repeatability of 91.6% in experimental results using 10 individuals' voice recordings with 50 repeated tests per each individual. The results showed that our method had better repeatability than the previous study. It proved that using features extracted from every valid frame was better than using averaged features of all valid frames. 

Averaged features could be good for monotonic utterance like vowels. However, features from the vowels are not proper to characterize individual voice quality. It can be easily realized that most of speaker identification applications use sentences, rather than vowels, since sentences are better to distinguish each individual's voice characteristics. Therefore, we conclude that detailed analysis using every single feature in each frame of the sentence, rather than using averaged features, is helpful to improve stability of SC diagnosis.

The proposed method is a text-dependent voice diagnosis, which uses the predefined sentence, and the same sentence must be used for both training and test stages equally. The stability will vary greatly when a different sentence is used in training and test stages. For future research, text-independent voice diagnosis method is required, so that subjects can speak freely to the system.

## Figures and Tables

**Table 1:** Mean values of *ts*_*j*_ in each Gaussian model.

No. of Gaussian	Female				Male		
	TE	SE	SY		TE	SE	SY
1	0.050	0.046	0.035		0.062	0.064	0.065
2	0.133	0.149	0.127		0.116	0.109	0.109
3	0.172	0.179	0.177		0.170	0.178	0.187
4	0.279	0.268	0.235		0.225	0.227	0.214
5	0.332	0.283	0.307		0.296	0.279	0.279
6	0.421	0.338	0.396		0.407	0.332	0.339
7	0.502	0.412	0.432		0.459	0.417	0.416
8	0.526	0.524	0.582		0.529	0.488	0.478
9	0.590	0.592	0.590		0.590	0.591	0.549
10	0.660	0.654	0.655		0.691	0.645	0.637
11	0.687	0.694	0.721		0.773	0.649	0.700
12	0.694	0.731	0.794		0.798	0.817	0.774
13	0.820	0.819	0.814		0.832	0.837	0.823
14	0.926	0.922	0.923		0.885	0.932	0.896
15	0.981	0.978	0.979		0.894	0.980	0.937

**Table 2:** Comparison results of diagnosis stability between the previous study and the proposed method.

Subject	Do et al. [4]				Proposed		
	Repeatability (%)	Probability mean	Probability standard deviation		Repeatability (%)	Probability mean	Probability standard deviation
1	68	0.561	0.098		100	0.460	0.016
2	76	0.632	0.119		100	0.434	0.039
3	100	0.732	0.090		98	0.406	0.027
4	54	0.500	0.059		100	0.397	0.025
5	98	0.735	0.112		100	0.451	0.031
6	100	0.672	0.090		70	0.381	0.020
7	84	0.603	0.100		82	0.362	0.013
8	56	0.567	0.082		76	0.381	0.013
9	70	0.689	0.130		90	0.384	0.019
10	76	0.629	0.140		100	0.408	0.017

Average	78.2	0.632	0.102		91.6	0.406	0.022

**Table 3:** Standard deviations of feature values from the sentence and vowels.

		Subject									
		1	2	3	4	5	6	7	8	9	10
Sentence features	MFCC1	0.290	1.287	0.535	0.414	0.333	0.317	0.432	0.269	0.395	0.245
	MFCC2	0.292	0.358	0.270	0.407	0.356	0.353	0.363	0.391	0.264	0.290
	MFCC3	0.325	0.597	0.302	0.350	0.635	0.413	0.511	0.205	0.313	0.259
	MFCC4	0.246	0.200	0.223	0.434	0.286	0.180	0.448	0.322	0.260	0.139
	MFCC5	0.451	0.320	0.352	0.423	0.479	0.309	0.708	0.470	0.288	0.280
	MFCC6	0.213	0.292	0.199	0.227	0.258	0.240	0.428	0.523	0.329	0.160
	MFCC7	0.336	0.287	0.428	0.266	0.341	0.294	0.311	0.278	0.262	0.307
	MFCC8	0.340	0.346	0.257	0.289	0.269	0.293	0.303	0.347	0.258	0.204
	MFCC9	0.268	0.399	0.279	0.521	0.374	0.307	0.480	0.420	0.362	0.211
	MFCC10	0.359	0.279	0.284	0.461	0.387	0.412	0.381	0.485	0.286	0.193
	MFCC11	0.251	0.334	0.304	0.412	0.375	0.411	0.432	0.410	0.251	0.235
	MFCC12	0.329	0.380	0.178	0.325	0.590	0.398	0.669	0.278	0.265	0.313

Vowel features	aENG	0.069	0.878	0.208	0.417	0.394	0.129	0.912	0.244	1.549	1.659
	aF1	0.634	0.336	0.453	0.380	0.282	0.552	0.489	0.652	0.366	0.346
	aSHIM	0.848	1.030	0.875	1.167	1.094	1.094	2.843	2.195	1.448	0.749
	eSHIM	1.127	1.397	0.986	1.555	0.873	1.120	2.870	3.048	1.393	0.541
	iDTF0	5.234	7.824	1.775	9.167	3.757	4.080	10.320	6.106	9.598	6.307
	iJITT	0.933	1.066	0.695	5.042	1.623	1.154	2.869	2.789	1.096	0.915
	oDTF0	2.330	6.399	1.296	3.616	3.767	3.623	9.513	8.807	1.316	1.178
	oPW	0.046	0.454	0.206	0.500	0.294	0.272	1.133	0.225	1.198	2.080
	uF1	0.263	0.286	0.874	0.580	0.188	0.356	0.422	0.273	0.216	0.659

Vowel features, xENG (energy), xF1 (1st formant), xSHIM (shimmer), xDTF0 (average difference of pitch over the time interval), xJITT (jitter), and xPW (power) were used in the study of Do et al. [4] x *∈*{a, e, i, o, u}.
